# Color and molecular structure alterations of brazilein extracted from *Caesalpinia sappan* L. under different pH and heating conditions

**DOI:** 10.1038/s41598-020-69189-3

**Published:** 2020-07-24

**Authors:** Luxsika Ngamwonglumlert, Sakamon Devahastin, Naphaporn Chiewchan, G. S. Vijaya Raghavan

**Affiliations:** 1 Advanced Food Processing Research Laboratory, Department of Food Engineering, Faculty of Engineering, King Mongkut’s University of Technology Thonburi, 126 Pracha u-tid Road, Tungkru, Bangkok, 10140 Thailand; 2 The Academy of Science, The Royal Society of Thailand, Dusit, Bangkok, 10300 Thailand; 3 Department of Bioresource Engineering, Faculty of Agricultural and Environmental Sciences, McGill University, 21111 Lakeshore Road, Ste. Anne de Bellevue, Quebec, H9X 3V9 Canada

**Keywords:** Chemical engineering, Sustainability

## Abstract

Brazilein extract from sappan wood (*Caesalpinia sappan* L.) has potential for use as natural food colorant since it has no unique flavor and taste. Although brazilein has long been applied in several traditional foods and beverages, information on its stability, which is of importance for practical application, is still limited. In this work, brazilein was isolated from sappan wood; its purity was confirmed by nuclear magnetic resonance spectroscopy. Relations between molecular structures and color as well as thermal stabilities of brazilein in aqueous solutions at pH 3, 7 and 9 were for the first time investigated. At the lowest pH, zero net-charge structure of brazilein, which exhibited yellow color, was predominantly found. The deprotonated and fully deprotonated structures of brazilein, which exhibited orange and red colors, respectively, were found when pH of the aqueous solutions increased. The forms of brazilein existing at the higher pH suffered extensive degradation upon heating, while the form existing at the lowest pH possessed higher stability. Heat-induced deprotonation and degradation were confirmed by UV–visible and Fourier-transform infrared spectra as well as losses of brazilein content.

## Introduction

Sappan (*Caesalpinia sappan* L.), which has several such local names as Buckham wood in English, Bois de sappan in French, Pau de sappan in Polish, Sappanholz in German, Su mu in Chinese, Suo in Japanese and Fang in Thai, is a wood in the family Leguminosae. This wood is naturally available throughout Asia, including China, Japan and Thailand. Due to its several valuable functions, this wood plant is also nowadays cultivated in various other regions of the world, including Africa, Europe as well as North and South Americas. Dried heartwood of sappan has long been used as herbal medicines as it contains many phytochemicals and bioactive compounds^1–5^. The biological abilities of the wood extract in terms of antiacne^6^, anti-bacterial^7,8^, anticancer^9^, antidiabetic^10^, antidiarrhea^11^, antiinflammatory^12–14^, antioxidant^15^ and anti-photoaging^16^ activities have been demonstrated by a number of researchers.

The major active ingredient of sappan is brazilin, which is a colorless phenolic compound consisting of two aromatic rings, one pyrone and one five-membered ring. Based on its molecular structure, brazilin is identified as a neoflavonoid. However, hydroxyl group in the brazilin structure is easily oxidized and can change into carbonyl group, resulting in the structural transformation and formation of brazilein, which is a colored compound^17–19^. Brazilein has indeed widely been used as a natural colorant; sappan extract containing brazilein has traditionally been used in the painting of books as well as in the dyeing of silk and wool. Since an aqueous extract of sappan possesses many beneficial properties over other red-color pigments, e.g., no unique flavor, no taste and inexpensiveness of the plant source, it has also been used as a colorant to yield red color in traditional foods and beverages in several Asian countries^4,20–24^.

To further extend the applicability of sappan extract as a natural food colorant, color stability, which is clearly related to molecular structure alteration, of the extract under various food processing conditions must be fully evaluated. Only a few existing works, however, focused on this important aspect. Among the limited available studies, Jin et al.^4^ assessed the use of sappan extract in pork sausage to reduce the use of synthetic additives (i.e., sodium ascorbate). Color and texture of the sausage added with the extract were reported and compared to those of the unadded one. However, the effect of food processing conditions (e.g., pH and heat) on the color stability was not studied.

Since brazilein is a polyphenolic compound, change in pH is expected to affect hydroxyl group in its molecule; such a structural change would in turn expectedly result in color change of the compound. Structural change of brazilein under various pH conditions was demonstrated via UV–VIS spectroscopy by Rondão et al.^19^. Djaeni et al.^25^ and Ulma et al.^26^ later reported that color of the aqueous sappan extract varied with pH. Under acidic condition, brazilein exhibited yellow color; the color shifted to red when pH increased into an alkaline region. However, the relations between visual color and molecular structures of brazilein at different pH have never been reported and discussed.

Besides pH change, heat, which is usually applied during food processing, is known to result in molecular structure change as well as degradation of natural pigments and hence change in their color^27^. Although the effect of heat on structural changes and/or degradation of several pigments, including, anthocyanins^28^, carotenoids^29,30^, betalains^31^ and chlorophylls^32,33^ has widely been demonstrated, study on the thermal stability of brazilein at different pH, which is related to its molecular structure, has never been conducted.

In this work, brazilein crystals were isolated from sappan wood; its purity was confirmed by nuclear magnetic resonance (NMR) spectroscopy. Color, in terms of lightness (L*), hue angle (h*) and chroma (C*), as well as molecular structure of brazilein in aqueous solutions at pH 3, 7 and 9 were then determined. Stability in terms of color changes, which are reported as the total color difference (∆E*), hue angle difference (∆h*) and chroma difference (∆C*), as well as molecular structure change of brazilein solutions at the different pH against heat at 60, 80 and 100 °C were for the first time investigated. The selected pH represents the use of brazilein at acidic, neutral and alkaline food processing conditions, while the selected heating temperatures refer to the use of the pigment at pasteurization and atmospheric boiling conditions. Change in the molecular structure was observed using UV–visible (UV–VIS) spectroscopy and Fourier-transform infrared (FTIR) spectroscopy. Brazilein content in the solutions was also measured via the use of high-performance liquid chromatography (HPLC) to quantify its degradation.

## Results and discussion

### Color and molecular structures of brazilein solutions at different pH

**Figure 1:**
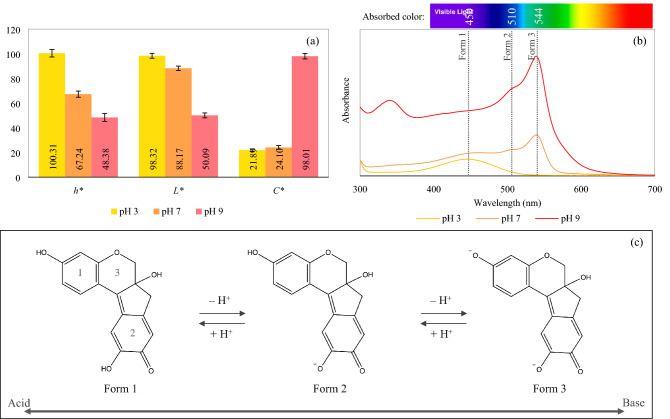
(**a**) Color values, (**b**) UV–VIS spectra and (**c**) possible predominant forms of brazilein at different pH.

Color values of brazilein aqueous solutions at different pH are shown in Fig. 1a. The results indicate limitation of the use of brazilein as a coloring agent due to the variation of its color upon pH change. The solution exhibited yellow hue (h* = 100.31°) at the lowest pH; increasing the pH to 7 and 9 resulted in the color shift towards orange hue (h* = 67.24°) and reddish hue (h* = 48.38°), respectively. Increasing the pH to the alkaline region also resulted in increased darkness and color saturation; the solution at pH 9 indeed exhibited the lowest L* and highest C* values.

The color results were related to the molecular structure of brazilein, which could be observed from the UV–VIS spectra. Three maximum absorbance wavelengths (λ_max_) at 450, 510 and 544 nm were noted as can be seen in Fig. 1b; these λ_max_ most probably represent the three existing forms of brazilein in the aqueous solutions. At the lowest pH value, brazilein form 1, which exhibits λ_max_ of 450 nm, was the predominant pigment in the solution. This form of brazilein exhibits yellow color. The solution at pH 7 (orange solution) consisted of brazilein form 1, form 2 (λ_max_ of 510 nm) and form 3 (λ_max_ of 544 nm), while the solution at pH 9 (red solution) mostly consisted of brazilein form 3.

Changes in the molecular structure of brazilein upon pH change might be due to protonation and deprotonation. Such changes are indeed common among other polyphenolic pigments. Hydroxyl (OH) group of polyphenolic compounds (e.g., anthocyanins and other flavonoids) is generally protonated under an acidic condition and deprotonated under an alkaline condition, resulting in molecular structure and hence color changes^34–36^. Although protonated and deprotonated structures of brazilein have been proposed by Rondão et al.^19^, the relationships between the different molecular structures and their color have never been reported. When these color and UV–VIS results were combined with the proposed brazilein structures, it could be summarized that brazilein form 1 should be the structure that possesses the net charge of zero. On the other hand, brazilein form 2 and form 3 should be the deprotonated and fully deprotonated structures, respectively, as shown in Fig. 1c. Note that these suggested form 2 and form 3 (or resonance forms) that exist at the higher pH are the structures of brazilein prior to heating. The structures of brazilein upon heating will be later presented.

The absorption intensity of O–H band of the freeze-dried powder prepared from the solutions at pH 7 and 9 should have decreased with increasing intensity of C=O band due to the transformation of the suggested brazilein form 1 into form 2 and/or form 3 through the process of deprotonation. However, no significant differences among the FTIR spectra of the freeze-dried powder prepared from the solutions at pH 3, 7 and 9 were observed (data not shown). This might be because the analyzed samples were in a powdery form; structural changes of brazilein under the acid–base conditions might take place only when the pigment existed in an aqueous form. However, even if the analysis was performed with aqueous solutions, both protonation and deprotonation of the OH group might still not be observed due to the high absorption intensity of the OH band of water overlaying the OH band of brazilein. In this work, FTIR spectroscopic analysis of a sample in an aqueous form was therefore not performed.

### Stabilities of brazilein solutions at different pH against heat

Combined effect of heat (at 60, 80 and 100 °C) and pH (at 3, 7 and 9) on changes in color and molecular structure of brazilein was investigated and is discussed in terms of color and molecular structure stabilities.

### Color stability

**Figure 2:**
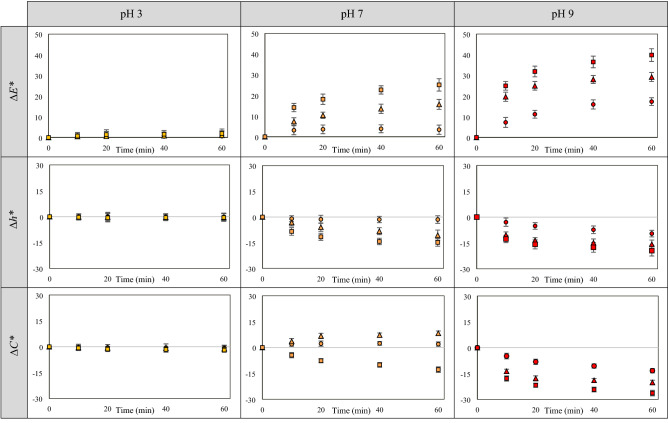
∆E*, ∆h* and ∆C* values of brazilein solutions at different pH heated at 60 °C (○), 80 °C (△) and 100 °C (□).

In all cases, increasing the pH as well as heating temperature and time led to increased ∆E*, ∆h* and ∆C* values; the results are shown in Fig. 2. All color values of the solution at pH 3 (yellow solution) did not significantly change upon heating at 60, 80 and 100 °C for 60 min; this implies the higher stability against heat of brazilein form 1, which predominantly exists in the solution at the lower pH (Fig. 1b,c). On the other hand, the color values of the solutions at pH 7 and 9 increased upon heating, indicating the lower stability of brazilein at the higher pH. Similar findings were noted in the cases of other polyphenolic pigments. Reyes and Cisneros-Zevallos^28^, Calogero et al.^36^, Fossen et al.^37^, Hurtado et al.^38^ and Jenshi roobha et al.^39^, for example, reported that flavylium cation, which is a predominant form of anthocyanins under an acidic condition (pH ≤ 3), possessed higher stability than the other forms, which exist at higher pH (e.g., quinoidal base, carbinol pseudobase and chalcone). Friedman and Jürgens^40^ also noted that increasing pH led to a decrease in the stability and to structural damage of plant phenolic compounds consisting of a large number of OH groups (e.g., chlorogenic acid and gallic acid). Nevertheless, structural stability of brazilein at different pH against heat has not so far been investigated.

Reduced stability at a higher pH in the case of phenolic compounds is noted to be due to the formation of quinone intermediate, which is easily degraded through oxidation reaction. Benzene ring in the phenolic structure that consists of hydroxyl groups located in ortho-position to each other, which is similar to ring No. 2 in the brazilein structure (Fig. 1c), can transform into quinone intermediate after deprotonation^40^. In this work, the deprotonated structures of brazilein (from 2 and/or from 3), which mostly existed in the solutions at pH 7 and 9, might transform into quinone intermediates; heat upon the stability test might later accelerate oxidation of such intermediates, resulting in color change as well as structural degradation, which was confirmed by the results on brazilein losses (data will be shown in the next section).

At neutral pH, color (in terms of ∆E*, ∆h* and ∆C*) of the orange solution only slightly changed upon heating at 60 °C for 60 min; color changes were more obvious when the heating temperature increased to 80 and 100 °C. Hue value of the solution at pH 7 heated at 80 and 100 °C decreased by about 10.64 ± 1.68° and 14.85 ± 1.42°, respectively. In terms of color saturation, chroma value of the solution at pH 7 heated at 80 °C surprisingly increased, while that of the same solution heated at 100 °C as well as that of the solution at pH 9 heated at 60, 80 and 100 °C significantly decreased. At pH 9, hue value also decreased by about 9.56 ± 0.89°, 15.73 ± 1.92° and 19.46 ± 1.74° after heating at 60, 80 and 100 °C for 60 min, respectively. The decrease in hue value represents an increase in redness of the solution. Among various stability test conditions, the most extensive change was observed in the case of the solution at pH 9 heated at 100 °C for 60 min; ∆E*, ∆h* and ∆C* values were 39.84 ± 2.48, − 19.46 ± 1.74° and − 26.35 ± 1.32, respectively.

Based on the aforementioned results, although color of the solution at pH 7 heated at 80 and 100 °C and of the solution at pH 9 heated at 60, 80 and 100 °C seemed redder than the unheated ones, only slight change in their hue after heating was observed by eyes. These color results were related to the molecular structure changes and/or degradation of brazilein upon heating, which will be discussed in the next section.

### Molecular structure stability

**Figure 3:**
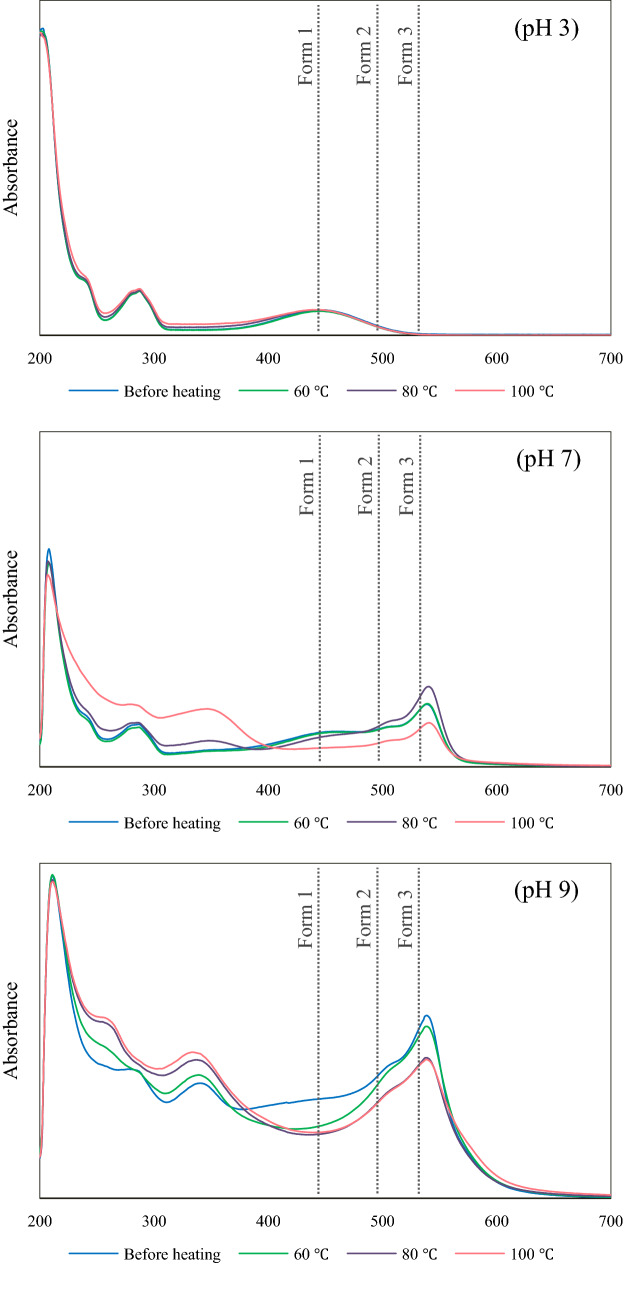
UV–VIS spectra of heated and unheated brazilein solutions at different pH.

Change in the molecular structure and degradation of brazilein were observed via UV–VIS spectra; the results are illustrated in Fig. 3. UV–VIS spectrum of the solution at pH 3 did not significantly change after heating; this result again confirms the higher stability of brazilein at acidic condition. At the higher pH, change in the molecular structure through deprotonation as well as structure degradation of brazilein upon heating were observed. As mentioned earlier, increasing pH led to deprotonation of OH group in the brazilein structure. The deprotonated structure could subsequently transform into quinone intermediate, which was easily degraded by heat-activated oxidation; the solution at the higher pH, which mostly contained the deprotonated structures of brazilein (form 2 and form 3), indeed suffered more extensive color change and brazilein degradation. At pH 9, the absorbance at 450, 510, 544 nm of the solution decreased after heating at 60, 80 and 100 °C for 60 min; the absorbance over the UV region at around 260, 290 and 345 nm, on the other hand, increased. Decreasing absorbance over the VIS region and increasing absorbance over the UV region indicated degradation of brazilein and formation of other colorless compounds, which might possibly be degradation products, respectively. The UV–VIS spectrum of the solution at pH 7 heated at 100 °C showed a similar trend to that of the heated solution at pH 9, while that of the solution at pH 7 heated at 80 °C exhibited a different trend.

After heating at 80 °C for 60 min, the absorbance at 450 nm of the solution at pH 7 slightly decreased; the absorbance at 510, 544 nm and over the UV region, in contrast, increased. In this case, change in the absorbance over the VIS region might be due to heat-induced change in the molecular structure of brazilein through deprotonation, leading to a decrease in the number of braziline form 1 as well as an increase in brazilein form 2 and form 3 as shown in Fig. 3. These structural changes well corresponded to the color results, i.e., an increase in redness and chroma after heating at 80 °C. Heat-induced deprotonation of phenolic compounds has also been reported in the case of anthocyanins^39^. On the other hand, an increase in the absorbance over the UV region should be due to the formation of colorless compounds (or degradation products); such a formation was quantified in terms of the percentage loss of brazilein (data will be shown in the next section). Although the degradation of brazilein was found in the case of the solution at pH 7 heated at 80 °C, a decrease in the absorbance at 510 and/or 544 nm was not observed. This is possibly because the rate of degradation might be lower than the rate of deprotonation. On the other hand, the degradation rate of the solution at pH 9 might be higher than the deprotonation rate as such a solution mostly contained the fully deprotonated brazilein. This led in turn to a decrease in the absorbance at 510 and 544 nm upon heating. Similar findings were noted in the cases of anthocyanins and other polyphenolic compounds; heat was noted to result in both degradation^41,42^ and change in the equilibrium of the four forms of anthocyanins^39^, resulting in visual color change.

**Figure 4:**
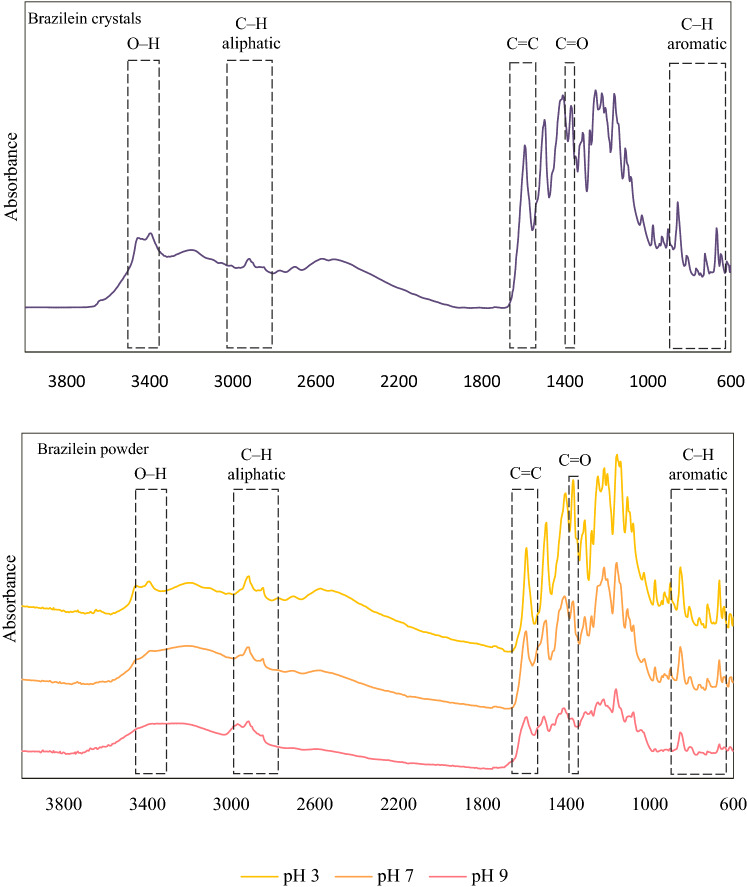
FTIR spectra of brazilein crystals and powder prepared from the solutions of different pH heated at 100 °C for 60 min.

Besides UV–VIS spectroscopy, the molecular structure change and degradation of brazilein at the higher pH were also confirmed via FTIR spectroscopy. FTIR spectra of brazilein powder prepared from the solutions at pH 3, 7 and 9 heated at 100 °C for 60 min were compared with those of brazilein crystals, which were isolated from the sappan extract; the results are shown in Fig. 4. After heating, FTIR spectrum of the powder prepared from the heated solution at pH 3 was similar to that of the brazilein crystals, while those of the powder prepared from the heated solutions at pH 7 and 9 showed significant differences. These observations agree with the unaltered color of the solution at pH 3 and the changing color of the solutions at pH 7 and 9 after heating at 100 °C for 60 min. The most extensive change was observed in the case of powder prepared from the heated solution at pH 9; this again confirms the lowest stability of brazilein at the alkaline condition or, in other words, the instability of the deprotonated forms of brazilein.

The absorbance intensity of OH group^3,43^ at around 3,425 and 3,380 cm^−1^ of the powder prepared from the solutions at pH 7 and 9 heated at 100 °C for 60 min significantly decreased, implying a decrease in the number of OH groups in the molecule, which might be due to heat-induced deprotonation. These results corresponded to the decrease in the number of brazilein form 1 and form 2 in the solutions at pH 7 and 9 heated at 100 °C (Fig. 3). Although the decrease in the number of OH group should have caused an increase in the intensity of C=O^3^ band at 1,365 cm^−1^, the results showed that the intensities of C=O as well as C=C^21,44^ bands at 1595 cm^−1^ and C–H band in the aromatic ring^3^ within the wavenumber range of 860–650 cm^−1^ in the spectra of the powder prepared from the solutions at pH 7 and 9 heated at 100 °C for 60 min decreased. Change in the pattern of aliphatic C–H band^43^ at around 2,980–2,845 cm^−1^ was also observed. These might be due to the degradation of the deprotonated brazilein (form 2 and/or form 3) after heating at a higher temperature for an extended period of time. These results corresponded to the decreased number of brazilein form 2 and form 3 in the solutions at pH 7 and 9 heated at 100 °C for 60 min (Fig. 3).

### Brazilein content

Degradation of brazilein upon heating was confirmed in terms of the percentage loss. Increasing pH, heating temperature and time led to an increase in the content loss as shown in Supplementary Fig. S1. The solution at pH 9 heated at 100 °C for 60 min suffered the most extensive loss of around 28.99 ± 1.32%; this corresponded to its most extensive changes in color, UV–VIS and FTIR spectra. At the lowest pH, only moderate losses (less than 10%) were observed upon heating at 60, 80 and 100 °C; such losses did not significantly affect the yellow color of the solution. In the case of the solution at pH 7, lower heating temperature (60 °C) only slightly affected the brazilein content as well as the orange color of the solution. On the other hand, losses of brazilein in the solution at pH 7 heated at 80 and 100 °C were 16.73 ± 1.24% and 23.19 ± 1.16%, respectively. Although this finding could be used to confirm the more extensive degradation (or losses) of brazilein at the higher pH upon heating, which seemed to be related to the molecular structure stability, the degradation mechanisms are still unclear and should be further elucidated.

## Conclusion

Brazilein crystals were isolated from sappan wood; purity of the crystals was confirmed by NMR spectroscopy. The relations between color and molecular structure as well as thermal stability of brazilein solutions at pH 3, 7 and 9 were then investigated; molecular structure changes were observed by UV–VIS and FTIR spectroscopy. Degradation of brazilein was also quantified. Brazilein was noted to be very sensitive to pH change as color of the solutions shifted from yellow to orange and red when the pH increased from 3 to 7 and 9, respectively. Change in color of the solutions at different pH was related to the molecular structure change via the process of deprotonation. At the higher pH, brazilein structure shifted toward the fully deprotonated structure; its structure, on the other hand, shifted backward to the zero net-charge form at the lower pH. The solution at pH 9 possessed the lowest thermal stability since the deprotonated structures existing in the solution might transform into quinone intermediate, which is easily degraded through oxidation. On the other hand, color and molecular structure of brazilein at pH 3 insignificantly changed upon heating. Both deprotonation and degradation of brazilein at the higher pH could be induced by heat; higher heating temperature therefore caused extensive color and molecular structure changes as well as brazilein degradation. Although the relations between color and molecular structure changes of brazilein upon heating were elucidated, the degradation mechanisms and formation of quinone or other intermediates should be further investigated. The obtained findings should nevertheless be useful when brazilein is to be used as a food colorant. Such findings are also necessary for further structural modification to enhance the pigment stability.

## Material and methods

### Plant material and reagents

Heartwood of sappan (*Caesalpinia sappan* L.) in a powdery form was purchased from Bang-Khae local market (Bangkok, Thailand). Taxonomic information of sappan is listed in the Integrated Taxonomic Information System (ITIS) report (taxonomic serial number: 506349).

Acetonitrile, ethanol and methanol were acquired from RCI Labscan (Samutsakorn, Thailand). Ammonium acetate, formic acid, hydrochloric acid and sodium hydroxide anhydrous were from Carlo Erba (Milan, Italy). All chemical reagents were of analytical grade.

### Brazilein extraction and isolation

Brazilein was extracted and isolated from sappan wood powder as per the suggested methods of Rondão et al.^19^ and Berger and Sicker^21^ with some modification. The processes started by mixing 300 g of the powder with 1,700 mL of pure ethanol. The mixture was continuously stirred for 3 days prior to being filtered through Whatman No. 1 filter paper. The clear solution (or extract) was later concentrated by a rotary evaporator (Buchi, R-215, Flawil, Switzerland) at 45 °C for 40 min. The final volume was adjusted to 100 mL with pure ethanol; the volume-adjusted extract was left at room temperature (~ 27 °C) for 6 days to crystallize brazilein. The formed black brazilein crystals were separated from the extract by subjecting the whole content to pressure-assisted filtration; the separated crystals were washed with cold methanol before being dried under vacuum (at an absolute pressure of 10 kPa) at 45 °C for 30 min to eliminate any remaining solvent prior to being kept in an opaque amber bottle.

To confirm the purity of the isolated brazilein, the crystals were dissolved in dimethyl sulfoxide-*d*_6_ (DMSO-*d*_6_) prior to the measurement of ^1^H NMR spectrum using a Fourier transform nuclear magnetic resonance spectrometer (FT-NMR) at 500 MHz (Bruker, Avance III™ HD, Karlsruhe, Germany). The obtained ^1^H NMR spectrum was in agreement with the spectrum of brazilein in DMSO-*d*_6_ reported by Kim et al.^45^ as listed in Supplementary Table S1.

### Stability evaluation

Stability (in terms of color and molecular structure changes) of brazilein in an aqueous solution at different heating and pH conditions was evaluated by first dissolving brazilein crystals in deionized water at the crystals to water ratio of 1:1,000 (w/v). pH of the solution was then adjusted to either 3, 7 or 9 with the addition of 0.1 M hydrochloric acid or 0.1 M sodium hydroxide solution. Color, UV–VIS and FTIR spectra of the solutions were determined.

Stability test was performed by filling 25 mL of each prepared solution into a series of screw cap test tubes, which were then submerged into a water bath (GFL, 1086, Burgwedel, Germany) at either 60, 80 or 100 °C. The sample tubes were removed from the bath after 10, 20, 40 or 60 min and suddenly cooled in an ice-water mixture. Note that pH of the heated solutions only slightly changed (less than 0.1) after each treatment. Color, UV–VIS and FTIR spectra as well as brazilein content of the heated samples were analyzed; the results were compared with those of the control sample, which is the unheated solution at similar pH conditions.

### Color measurement

Color of a test solution was measured in the CIELAB color space (L*, a* and b*) using a spectrophotometer (HunterLab, ColorQuest XE, Reston, VA, USA). The measurement was taken in a transmittance mode using a simulated D65 illuminant and an observer angle of 10°. Changes in the color of the solutions at different pH conditions upon heating are reported in terms of chroma difference (∆C*), hue angle difference (∆h*) and total color difference (∆E*); these parameters were calculated as per Eqs. (1)–(5).

1 $$Chroma \left({C}^{*}\right)=\sqrt{{a}^{*2}+ {b}^{*2}}$$

2 $$Chroma\,difference \left(\Delta {C}^{*}\right)=\left|{C}_{i}^{*}-{C}_{0}^{*}\right|$$

3 $$Hue\,angle \left({h}^{*}\right)={tan}^{-1}({b}^{*}/{a}^{*})$$

4 $$Hue\,angle\,difference \left(\Delta {h}^{*}\right)=\left|{h}_{i}^{*}-{h}_{0}^{*}\right|$$

5 $$Total\,color\,difference \left({\Delta E}^{*}\right)=\sqrt{{\left({L}_{i}^{*}-{L}_{0}^{*}\right)}^{2}+{\left({a}_{i}^{*}-{a}_{0}^{*}\right)}^{2}+{\left({b}_{i}^{*}-{b}_{0}^{*}\right)}^{2}}$$

where L*, a* and b* are lightness, redness and yellowness of a solution, respectively. The subscripts i and 0 represent a sample under heating at any instant and an unheated one (control sample), respectively.

### Molecular structure change determination

Changes in the molecular structure of brazilein upon pH adjustment and heating were determined by UV–VIS and FTIR spectroscopies. To obtain UV–VIS spectra, both unheated and heated brazilein aqueous solutions at different pH were scanned over the wavelength range of 300–700 nm via the use of a spectrophotometer (Shimadzu, UV-2600, Kyoto, Japan).

For FTIR spectra determination, each solution was firstly dried in a freeze dryer (Martin Christ, Alpha 1-4 LSCplus, Osterode am Harz, Germany). The obtained powder was introduced to an FTIR spectrometer (Perkin Elmer, Spectrum One, Waltham, MA, USA) using 10 or 2 scans at 4 cm^−1^ spectral resolution over the wavenumber range of 4,000–400 cm^−1^.

### Brazilein content determination

Brazilein content of a solution was analyzed according to the recommended methods of Zhao et al.^46^ with some modification. Around 20 mL of the solution was freeze dried and then reconstituted by the addition of 2 mL of pure ethanol. The ethanol-based sample was filtered through a syringe filter nylon membrane prior to being injected into a high-performance liquid chromatography (HPLC) system.

Agilent 1100 Series HPLC system consisting of a G1397A degasser, a G1311A quaternary pump, a G1313A standard autosampler, a G1316A thermostatted column compartment (TCC) and a G1314A variable wavelength detector (VWD) was used to determine the brazilein content. The sample volume of 10 µL was injected into a Supelco C18 (5 µm) HPLC column (4.6 × 250 mm) (Merck KGaA, Darmstadt, Germany) using 30% (v/v) acetonitrile containing 0.1% (v/v) formic acid and 10 mM ammonium acetate as a mobile phase at a flow rate of 0.6 mL/min. The column temperature and the detection wavelength were set at 25 °C and 445 nm, respectively.

After a small volume (10 µL) of the sample solution at different pH was injected into the HPLC system, the final pH of such a sample should reach an acidic value of the mobile phase. The presented form of brazilein in the HPLC system should therefore be the form that exists at acidic condition, which can be detected at 445 nm. The retention time was around 12 min (Supplementary Fig. S2a). The brazilein content was calculated from the brazilein standard curve (Supplementary Fig. S2b) prepared by dissolving the isolated brazilein crystals in pure ethanol at concentrations of 20–100 ppm. Note that the high purity of the isolated crystals should be ensured as the chromatogram showed only one peak of brazilein (Supplementary Fig. S2a). ^1^H NMR spectrum of the crystals was also in excellent agreement with that of brazilein reported by Kim et al.^45^ (Supplementary Table S1). The content is reported in terms of percent loss, which was calculated using Eq. (6).

6 $$\% Brazilein\,loss=(({B}_{0}-{B}_{i})/{B}_{0})\times 100$$

where *B*_i_ and* B*_0_ are brazilein content of a sample heated at any instant and that of an unheated one (control sample), respectively.

### Statistical analysis

All experiments were performed at least in triplicate and the obtained data were subject to the analysis of variance (ANOVA) using a statistical program Minitab (version 16). All results are presented as mean values with standard deviations. To establish differences among mean values, least significant difference (LSD) tests at a confidence level of 95% were conducted.

## Supplementary information

Supplementary information

## Data Availability

Experimental and relevant data are available from the authors upon request.
