# Supplementary material for: Color and molecular structure alterations of brazilein extracted from Caesalpinia sappan L. under different pH and heating conditions
Source: Sci Rep. 2020 Jul 24;10:12386. doi: 10.1038/s41598-020-69189-3 (PMC7382456; doi:10.1038/s41598-020-69189-3)
Supplement: Supplementary file 1 — Supplementary information [file 41598_2020_69189_MOESM1_ESM.docx]

**Color and molecular structure alterations of brazilein extracted from *Caesalpinia sappan* L. under different pH and heating conditions**

**Luxsika Ngamwonglumlert^1^, Sakamon Devahastin^1,2,^*, Naphaporn Chiewchan^1^, and G. S. Vijaya Raghavan^3^**

^1^Advanced Food Processing Research Laboratory, Department of Food Engineering, Faculty of Engineering, King Mongkut’s University of Technology Thonburi, 126 Pracha u-tid Road, Tungkru, Bangkok, 10140, Thailand

^2^The Academy of Science, The Royal Society of Thailand, Dusit, Bangkok 10300, Thailand

^3^Department of Bioresource Engineering, Faculty of Agricultural and Environmental Sciences, McGill University, 21111 Lakeshore Road, Ste. Anne de Bellevue, Québec, Canada H9X 3V9

*Corresponding author.

Tel.: +66 2 470 9244; Fax: +66 2 470 9240

E-mail: sakamon.dev@kmutt.ac.th

Co-authors: Luxsika Ngamwonglumlert (solu_ping@yahoo.com); Naphaporn Chiewchan (naphaporn.rat@kmutt.ac.th); G. S. Vijaya Raghavan (vijaya.raghavan@mcgill.ca)

**Supplementary Table S1** ^1^H NMR of brazilein dissolved in DMSO-*d*_6_

| 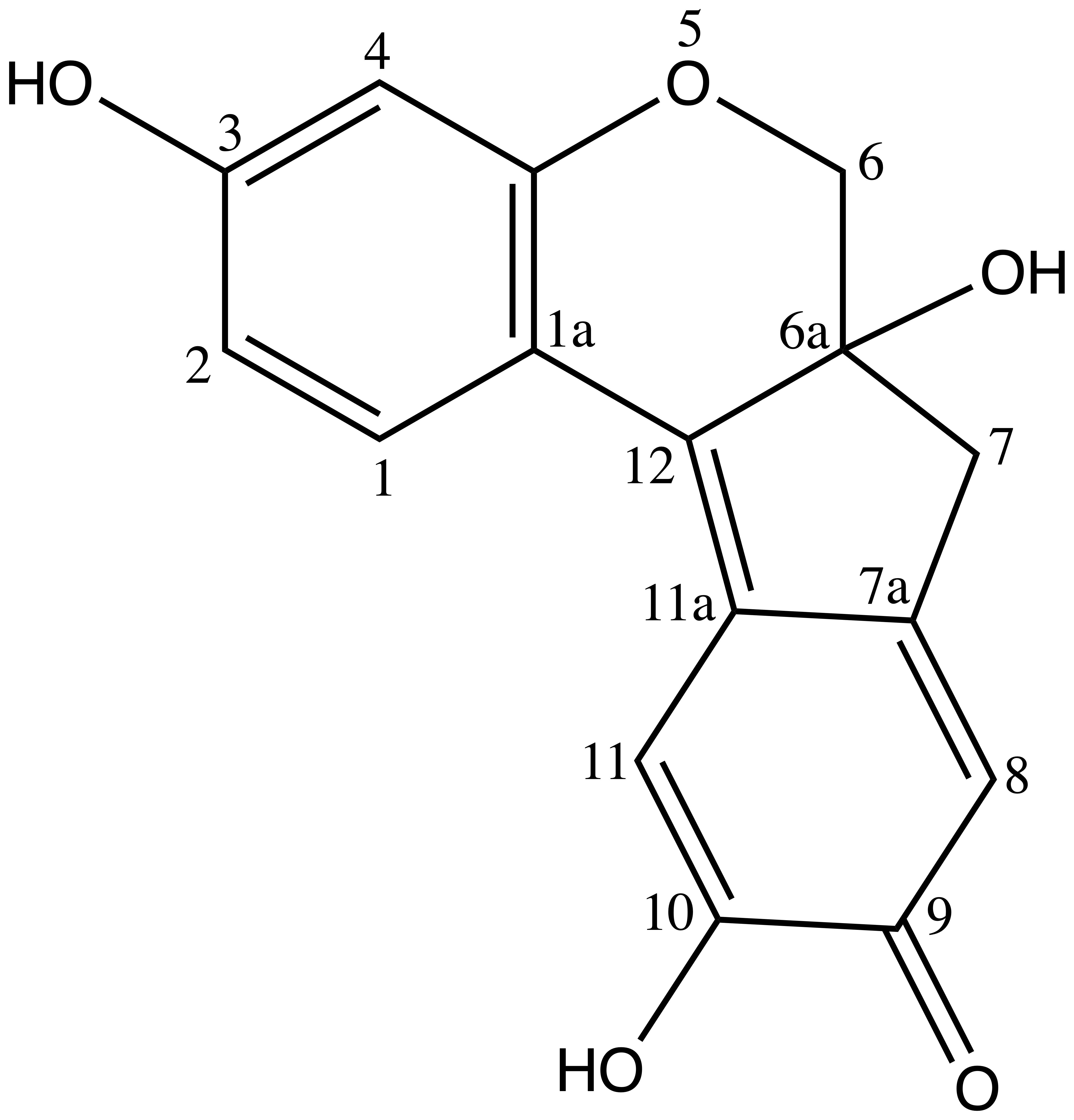  Brazilein structure^44–45^ | | |
| --- | --- | --- |
| Position | δ_H_ (ppm) (Nh, m), *J* (Hz) | |
|  | Kim et al.^45^ | Present work |
| 1 | 7.91 (1H, *d*), *J* = 8.8 | 7.80 (1H, *d*), *J* = 8.8 |
| 2 | 6.67 (1H, *dd*), *J* = 8.8, 2.3 | 6.54 (1H, *dd*), *J* = 8.8, 2.3 |
| 4 | 6.47 (1H, *d*), *J* = 2.3 | 6.35 (1H, *d*), *J* = 2.3 |
| 6 | 4.10 (1H, *d*), *J* = 11.8 | 4.00 (1H, *d*), *J* = 11.6 |
|  | 4.56 (1H, *d*), *J* = 11.8 | 4.46 (1H, *d*), *J* = 11.6 |
| 7 | 2.95 (2H, *s*) | 2.84 (2H, *s*) |
| 8 | 6.43 (1H, *s*) | 6.31 (1H, *s*) |
| 11 | 7.22 (1H, *s*) | 7.09 (1H, *s*) |

δ = Chemical shift; H = Amount of proton; *dd* = Doublet of doublets; *d* = Doublet; *s* = Singlet; *J* = Coupling constants

(pH 9)

(pH 7)

(pH 3)

**Supplementary Figure S1** Percent brazilein losses at different pH after heating at 60 °C (○), 80 °C (△) and 100 °C (□).


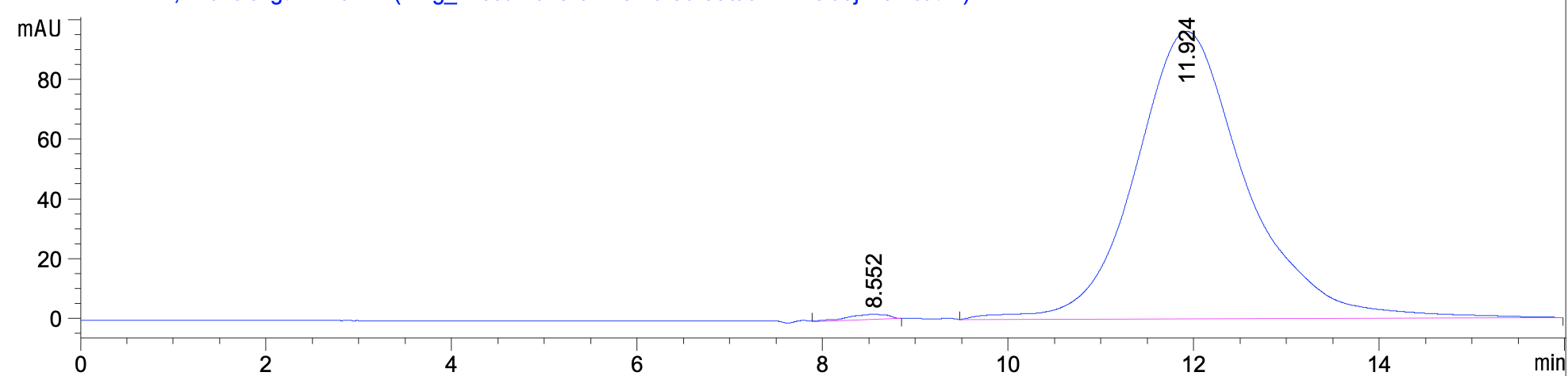


min

(a)

0

2

4

6

8

10

12

14

mAU

80

60

40

20

0


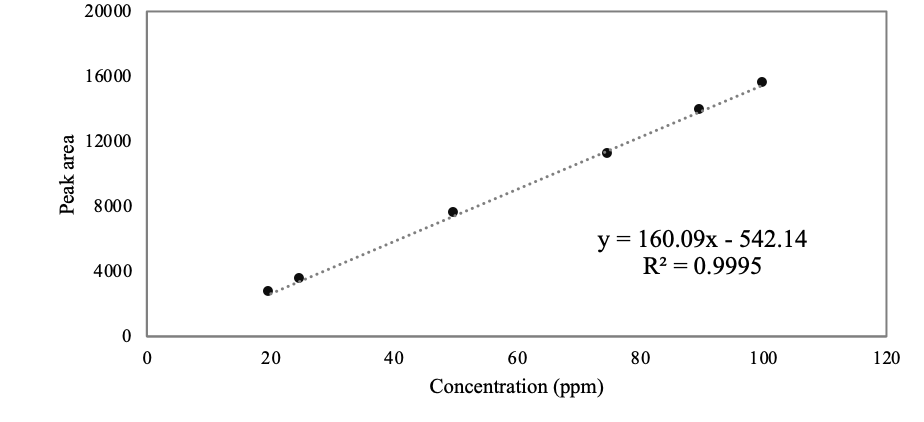


(b)

**Supplementary Figure S2** (a) HPLC chromatogram and (b) standard curve of brazilein.
